# Chronic helminth infection burden differentially affects haematopoietic cell development while ageing selectively impairs adaptive responses to infection

**DOI:** 10.1038/s41598-018-22083-5

**Published:** 2018-02-28

**Authors:** Simon A. Babayan, Amy Sinclair, Jessica S. Duprez, Colin Selman

**Affiliations:** 1 Institute of Biodiversity, Animal Health & Comparative Medicine, University of Glasgow, Glasgow, UK; 2 Moredun Research Institute, Pentlands Science Park, Penicuik, UK; 3 Glasgow Ageing Research Network (GARNER), Institute of Biodiversity, Animal Health & Comparative Medicine, University of Glasgow, Glasgow, UK; 4 School of Biomedical Sciences, University of Edinburgh, Edinburgh, UK

## Abstract

Throughout the lifespan of an individual, the immune system undergoes complex changes while facing novel and chronic infections. Helminths, which infect over one billion people and impose heavy livestock productivity losses, typically cause chronic infections by avoiding and suppressing host immunity. Yet, how age affects immune responses to lifelong parasitic infection is poorly understood. To disentangle the processes involved, we employed supervised statistical learning techniques to identify which factors among haematopoietic stem and progenitor cells (HSPC), and both innate and adaptive responses regulate parasite burdens and how they are affected by host age. Older mice harboured greater numbers of the parasites’ offspring than younger mice. Protective immune responses that did not vary with age were dominated by HSPC, while ageing specifically eroded adaptive immunity, with reduced numbers of naïve T cells, poor T cell responsiveness to parasites, and impaired antibody production. We identified immune factors consistent with previously-reported immune responses to helminths, and also revealed novel interactions between helminths and HSPC maturation. Our approach thus allowed disentangling the concurrent effects of ageing and infection across the full maturation cycle of the immune response and highlights the potential of such approaches to improve understanding of the immune system within the whole organism.

## Introduction

As individuals age, their immune system deteriorates — a process termed immunosenescence. This is characterised by a general disruption of immune homeostasis, including the impaired immune cell development in the bone marrow, thymic involution, increased risk of autoimmunity, weaker responses to new and chronic infections, and attenuated responses to vaccination^1–6^. However, our understanding of how ageing specifically affects the host’s ability to mount and maintain protective immunity to infection is hampered by the range and complexity of the processes involved^7,8^. Furthermore, little is known about how ageing affects long-term adaptive immune responses to chronic infections, the most wide-spread of which are caused by parasitic helminths that, in turn, profoundly affect their hosts’ immune system^9,10^.

Typically, research into complex host-parasite interactions has relied on studying ageing and infection in isolation, rather than investigating how multiple immune components interact in the context of ageing and infection — limitations imposed, in part, by the statistical tools available. As high throughput platforms become the norm and increasingly yield compelling insight, methods for the analysis of large complex biological datasets are diversifying^11,12^. Machine learning approaches are proving informative for identifying meaningful features within data sets containing an increasing number of variables, and can perform well both for predictive and mechanistic inference even under the relatively low sample/feature size ratios typical of biomedical research^13–15^. Such approaches have been successfully applied to immunology and to ageing, for example, in predicting lifespan using morphometric and immune markers^16,17^, and in identifying markers of immune senescence^18^. Here we applied such algorithms to help elucidate, throughout the main developmental lineages of the immune system, how ageing and chronic infection interact by using well-established models of infection under controlled settings. Specifically, we aimed to identify which haematopoietic stem and progenitor cells (HSPC), immune cells, and cytokines were more robustly associated with protective immunity to a chronic helminth infection, and determine how age-associated deterioration of the immune system affected those features. Using the filarial parasite *Litomosoides sigmodontis*^19^, we infected 2-month-old and 10-month-old naive female mice, allowed their parasites to mature and reproduce for 2 months and then compared their immune profiles to age-matched uninfected controls. We then necropsied the then 4-month-old (4mo) and 12-month-old (12mo) mice, and measured 79 immune variables spanning HSPC to mature cell types, in addition to parasite survival and the relative production of transmissible offspring, the microfilariae. We used supervised machine learning to aid our identification of the immune factors that predicted adult worm and microfilariae burdens by iteratively validating model predictions using different randomised test sets (nested cross-validation) drawn from the full dataset to maximise our confidence in the associations found, and then assessed how host age affects their expression in relation to parasite burden.

## Results and Discussion

### Ageing increased susceptibility to *Litomosoides sigmodontis*

**Figure 1:**
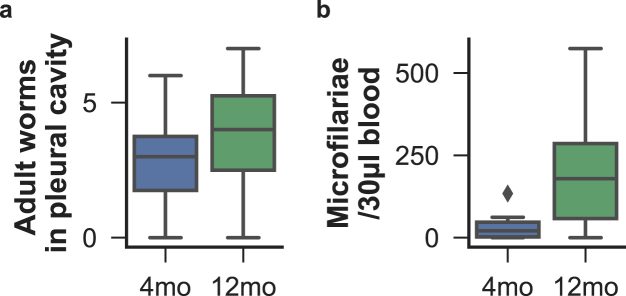
Susceptibility to helminth infection increased with age. Parasite survival and fecundity were measured in 4 and 12-month-old (respectively 4mo and 12mo) mice that were subcutaneously infected 2 months prior with 40 *L*. *sigmodontis* infective larvae to investigate the effects of host age on susceptibility to chronic helminth infection. (**a**) While no significant difference was observed in adult parasite survival between the different age classes, (**b**) greater densities of microfilariae were detected in the peripheral blood of 12mo than of 4mo mice (P_Mann-Whitney_ = 0.038, n = 8 per group). Worm counts are summarised as boxplots where horizontal lines represent the group median, boxes the inter-quartile range, whiskers the overall range, and diamonds represent outliers (<± 1.5 × the interquartile range).

Filarial parasites are extremely resilient to host immune responses, as shown by their ability to maintain chronic infections even after vaccination^20–23^. However, the filarial life stages most sensitive to immune attack are their offspring, the microfilariae, as shown by the reduction or even elimination of parasite fecundity in vaccinated animals and in resistant strains^24,25^. Here, we examined how age affected parasite survival in wild type BALB/c mice, which are normally susceptible to *L*. *sigmodontis* infection^19^. While parasite survival was unaffected by host age (Fig. 1a) as typical of primary infection with this model^26,27^, we found that parasite fitness, determined by measuring the concentration of microfilariae in the host’s peripheral blood, was significantly higher in 12mo relative to 4mo mice (Fig. 1b). This increased susceptibility to parasites is consistent with previous reports of viral, bacterial, fungal, and helminth infection burdens which increase with age in multiple host species^28–32^. Our results suggest there may be distinct processes involved in limiting parasite establishment and parasite fecundity, and that those resistance mechanisms may be differentially affected by ageing. To disentangle the immunological changes that occur with age, we sought to identify (i) the HSPC and mature immune cells that differed between mouse age classes, (ii) the immune factors that best predicted adult worm burdens and microfilariae densities, and (iii) which among those predictors were affected by host age.

### Ageing affected naive and memory T cell populations systemically but not HSPC bone marrow populations

**Figure 2:**
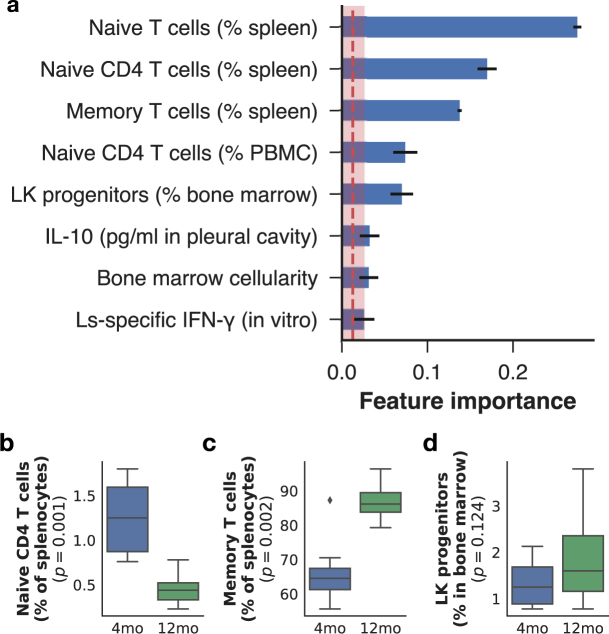
Naive and memory T cell proportions were the main differentiator between 4mo and 12mo mice. The importance of each immune factor in predicting host age was estimated by measuring the average gain in the purity of daughter nodes when decision tree splits used that feature for classifying all 32 mice mice as young or old irrespective of their infection status. Thus, the greater the importance of an immune feature, the more useful it is in classifying a mouse as 4mo or 12mo. We used a cut-off of 3 standard errors (red-shaded zone) above the mean (red dotted line) of all importances below which features were not considered robust enough for further inference. (**a**) Top predictors of age in mice, whether infected or not, achieved 97% accuracy on average over 10 repeated random training sets in correctly clarifying mice as 4mo or 12mo. Horizontal bars represent means and associated errors of the feature importances generated from 10 models trained on different nested random 75%/25% train/test repeats. Top predictors included the proportions of naive and memory T cells in the spleen as expected under thymic involution (see Supplementary Fig. S3): in the spleen, (**b**) the proportion of naive T helper cells (CD3+ CD4+ ) had decreased, whereas (**c**) the proportions of memory T cells exceeded 80% of all splenic T cells in 12mo mice while ranging between 55% and 72% in 4mo mice. (**d**) Among HSPC within the bone marrow, Lin^−^c-Kit^+^Sca-1^−^ (LK) cells were found in greater proportions in 12mo than in 4mo mice. See methods section for details on feature importance calculations. Horizontal bars represent means and associated errors of the feature importances generated from models trained on and testing against 10 repeated 75%/25% random splits of the full dataset. In boxplots, horizontal lines represent the group median, boxes the interquartile range, whiskers the overall range, and points represent outliers (<± 1.5 × the interquartile range, n = 16 per mice class).

Immunosenescence is associated with widespread changes in the composition and responsiveness of the immune system. We first assessed whether the 79 immune variables we measured differed between age classes without prior knowledge of the true age of the animals. We reduced the immune features to two components using a principal component analysis (PCA), and used k-means clustering to identify how mice clustered along those two dimensions. This resulted in a decision boundary that separated most of mice according to their age, suggesting tractable differences in the composition of the immune system between the two age groups, irrespective of their infection status (Supplementary Fig. S1). To identify which of those immune factors were most robustly associated with host age, we took a supervised learning approach and trained gradient boosted trees^33^, which were projected to be the best performing algorithm for this task based on baseline algorithm performance (Supplementary Fig. S2a), on 79 immune factors spanning immune cell development in multiple organs (see Supplementary methods). Gradient boosting is a highly effective machine learning technique that combines several weak learners, or models, which need perform only slightly better than random chance to create a strong “consensus” prediction. We used XGBoost^34^, a flexible implementation of gradient boosting machines, to predict host age class (Fig. 2). The models were trained on immune features and mouse age class on all animals irrespective of infection status using repeated randomised nested sampling of 75% of the mice, and achieved a median 100% accuracy in correctly classifying the remaining “test” mice that were retained for model testing, and revealed several strong predictors of age class (Fig. 2a). The most important factors for prediction accuracy were consistent with known effects of immune senescence — namely, the decreased proportions of naive T cells and increased proportion of memory T cells, typical of thymic involution^35^ (Fig. 2b,c, and see Supplementary Fig. S3 for further evidence of thymic involution). The trained model also revealed signatures of host age on Lin^−^Sca-1^+^c-Kit^−^ (LK) cells, which were increased in the bone marrow of 12mo compared to that of 4mo mice (Fig. 2d). Thus, at the ages we examined, despite significant differences in T cells, HPSC only marginally differed between age classes.

### Adult worm burdens were negatively associated with HSPC proportions

**Figure 3:**
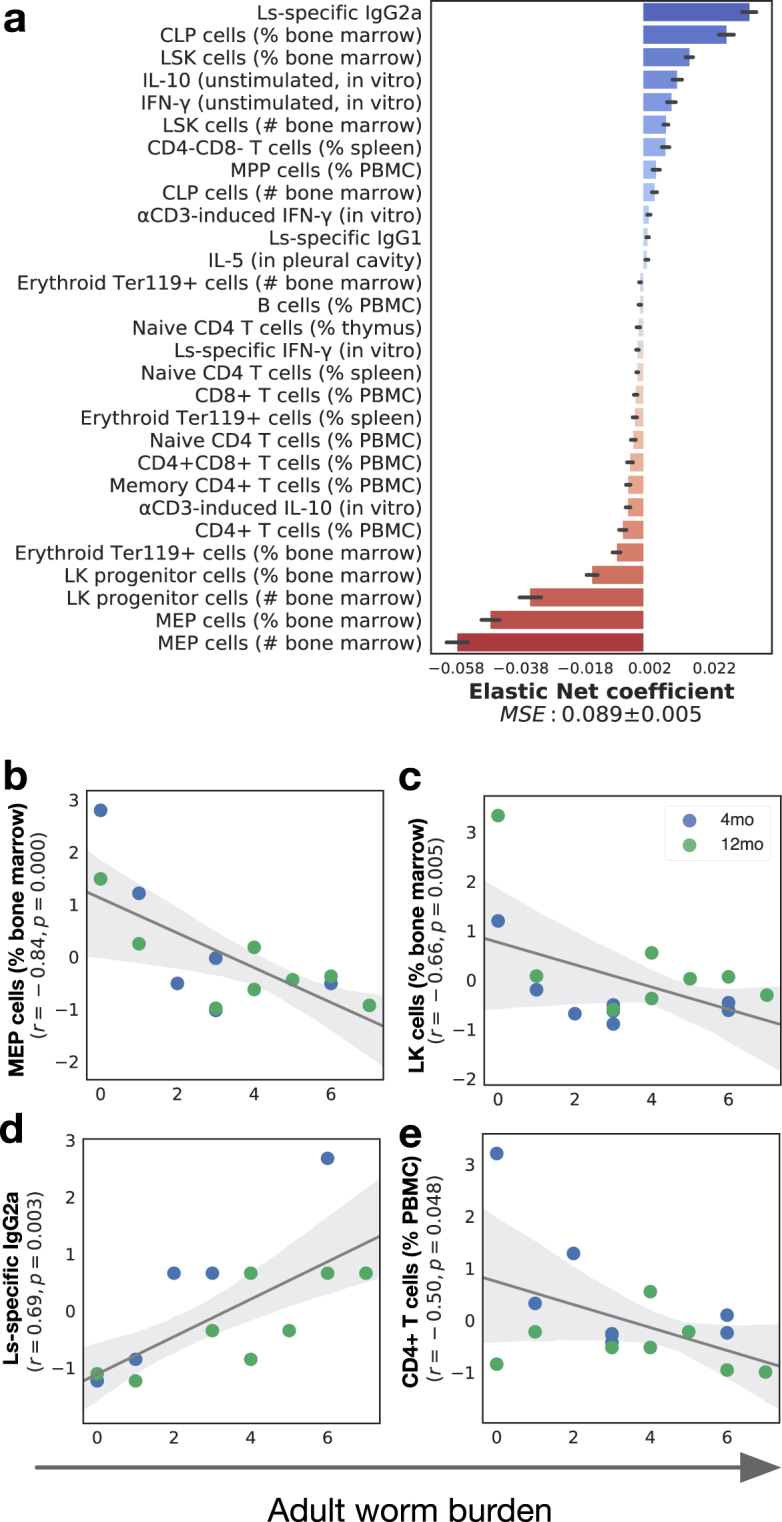
Adult worm burden was negatively correlated with the abundance of MEP, LK, and CD4 T cells, but positively associated with parasite-specific IgG2a titres. (**a**) Elastic Nets were used to map immune features to adult worm burdens 2 months post infection in infected mice only (n = 16). A wide range of cell types, comprising HSPC, innate, and adaptive immune cells, contributed to correctly predicting adult burdens with a mean squared error of 0.089 ± 0.005: the strongest predictors of worm burden among HSPC included (**b**) the proportions of MEP (r = −0.84; P ≤ 0.001, GLM), (**c**) LK cells, both negatively associated with parasite burdens, and from the peripheral blood circulation (r = −0.66; P = 0.005, GLM), (**d**) the titre of Ls-specific IgG2a (r = 0.69; P = 0.003, GLM) and (**e**) the proportion of CD4+ T cells among PBMC (r = 0.50; P = 0.048, GLM). Horizontal bars represent means and associated errors of the coefficients applied by the Elastic Net models trained on 10 repeated 75% / 25% random nested splits of the full dataset. In regression plots, each point represents a mouse, the solid line is the robust regression (preferred over least squares to reduce the influence of outliers) and the shaded areas represent the corresponding 95% bootstrapped confidence intervals.

The effects of infection on the overall set of immune variables, as examined by an unsupervised PCA, showed weak separation between infected and uninfected animals, suggesting a supervised approach is necessary (Supplementary Fig. S1). XGBoost identified Ls-specific IgG1 in the blood as one of the best predictors of the presence of infection by *L*. *sigmodontis* (Supplementary Fig. S4a,b), which together with B lymphocyte and eosinophil percentages among pleural exudate cells (PLEC) as well as IL-4 and IL-5 production (Supplementary Fig. S4c–e), enabled models to reach 100% median accuracy in classifying mice as infected or not (Supplementary Fig. S4g). However, identifying protective immune mechanisms in individuals exposed to infection requires examining the quantitative relationships between immune factors and parasite survival and/or fecundity. A comparison between five common regression algorithms (linear regression, Elastic Nets, k nearest neighbour, random forests, and extreme gradient boosting) suggested that Elastic Nets^36^ would be better suited for this task (Supplementary Fig. S2c). We therefore trained an Elastic Net to identify the best correlates of adult worm burden, which achieved a mean square error of 0.089 ± 0.005 (Fig. 3a). Then for each of the best immune correlates, we tested whether host age affected the response to each parasite life stage using generalised linear models.

Considering only infected animals, worm burden was associated with systemic cellular changes that spanned HSPC, innate, and adaptive immune factors, to which the Elastic Nets allocated both postive and negative weights (Fig. 3). Positive associations between immune factors and parasite burdens may suggest dose-responses to infection, while negative associations can either denote protective immunity (i.e. the immune factor kills worms) or active suppression by the parasites — unless previously reported, disentangling these effects would require experiments (e.g. genetic modification) that lie beyond the scope of this study. Strikingly, MEP total numbers in bone marrow and proportions among HSPC consistently ranked as strongest negative correlates of adult worm burdens (Fig. 3a,b). This is notable for three reasons: first, megakaryocytes produce inflammatory mediators that recruit immune cells and may thus directly contribute to protection against helminth infection; second, they produce platelets, an important element of wound healing^37,38^, which appears consistent with the hypothesis that wound healing processes and/or clotting are an integral part of responses to tissue-dwelling helminths^39–41^; conversely, *L*. *sigmodontis* may actively suppress MEP differentiation, consistent with reports of nematodes suppressing clotting, a process driven by platelets^42–45^ although to our knowledge, direct suppression of MEP cells by parasites has not previously been reported.

While Elastic Nets utilise the predictive power of all features in aggregate, we wished to further identify those that correlated significantly with worm burdens in isolation. This procedure highlighted the strong associations between worm burden and MEP cells, LK cells, and Ls-specific IgG2a (Fig. 3b,c,d) as well as a weaker positive association with CD4 T cells (Fig. 3e). However, factors previously associated with protection against *L*. *sigmodontis*, such as IgG1, IL-5, and eosinophils, only weakly contributed to predicting parasite burdens, and did not reach statistical significance on their own despite being strong markers of the presence of an infection (Supplementary Fig. S4a–e). This is consistent with our previous studies of infections at these time points, in which those factors only correlated with parasite burdens when projected onto a principal component but not when examined in isolation^21^, despite them being necessary for protection^46–48^.

### Microfilariae densities were predominantly associated with inflammatory and adaptive immune responses

**Figure 4:**
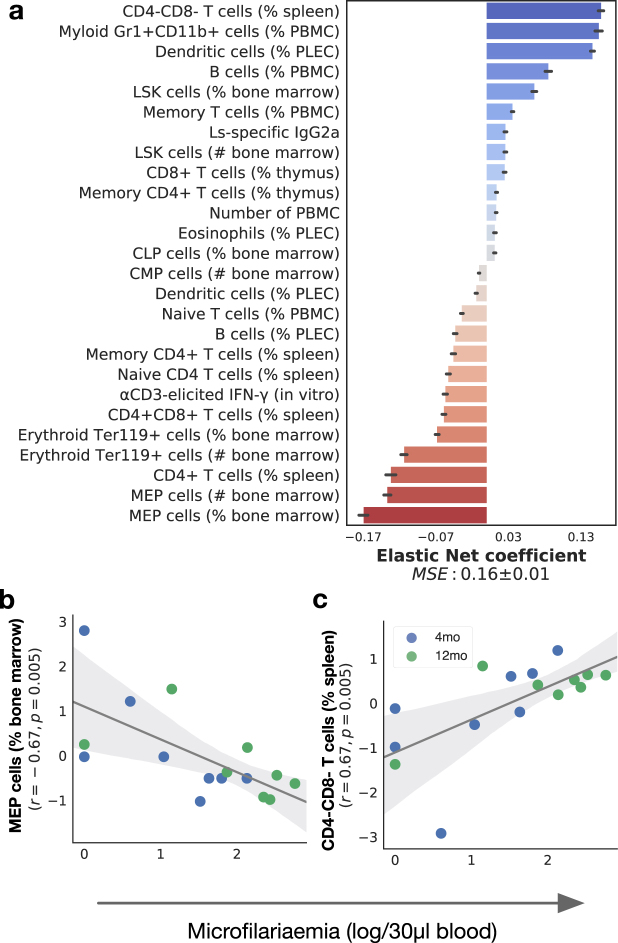
Microfilarial densities were positively correlated with the proportions of CD4-CD8- T cells in the spleen but negatively correlated with MEP cell proportions in the bone marrow. (**a**) The importance of each immune factor in predicting the density of blood-circulating microfilariae 2 months post infection in infected mice only (n = 16) was estimated using Elastic Nets, which achieved an average MSE of 0.16 ± 0.01 (see methods for details). Among the immune factors that best predicted microfilaraemia, those that were significantly correlated with microfilariaemia in isolation included the proportions of (**b**) MEP cells in the bone marrow (r = −0.67; P = 0.005, GLM) and (**c**) the percentage of CD4−CD8− T cells in the spleen (r = 0.67; P = 0.005, GLM). Horizontal bars represent means and associated errors of the coefficients applied by the Elastic Net models trained on 10 repeated 75%/25% random splits of the full dataset. In regression plots, each point represents a mouse, the solid line is the robust regression (preferred over least squares to reduce the influence of outliers) and the shaded areas represent the corresponding 95% bootstrapped confidence intervals.

**Figure 5:**
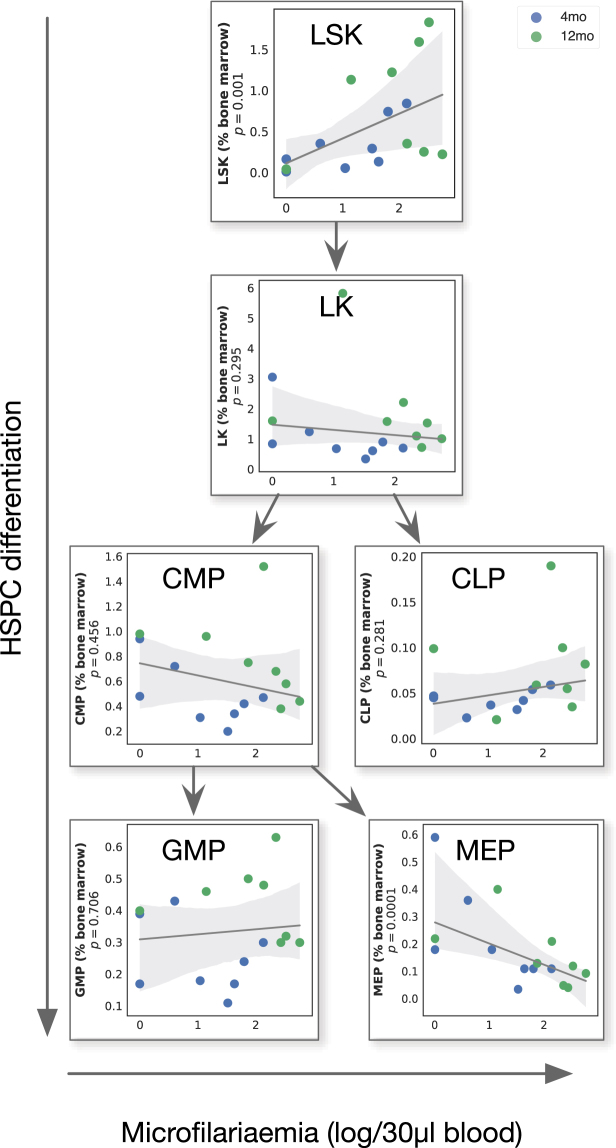
Association between worm burdens and HSPC proportions through haematopoietic development. Robust regression between microfilaraemia and percentage of HSPC in the bone marrow through haematopoietic maturation. Each point represents a single mouse (both ages combined) and the shaded areas represent the 95% bootstrapped confidence intervals around the robust regression line. P values from GLM of main effect of log-transformed microfilariae counts on the described cell type (n = 16).

Considering only infected animals as above, worm fecundity (assessed by the density of blood-borne microfilariae) was also associated with systemic cellular changes, and spanned HSPC, innate, and adaptive immune factors as identified by an Elastic Net achieving a mean square error of 0.16 ± 0.01. As with adult worm burdens, MEP total numbers and proportions consistently ranked as strongest negative correlates of microfilariae densities (Fig. 4a,b). Immune factors that contributed most strongly to robust prediction of microfilariae densities included positively-associated proportions of CD4−CD8− T cells in the spleen, myeloid Gr1+ CD11b+ cells and B cells in the blood, and dendritic cells in the pleural cavity. The negatively-associated factors included erythroid Ter119 + and MEP cells in the bone marrow, and the proportion of CD4+ T cells in the spleen. Taken in isolation, predictors that significantly correlated with microfilariae densities comprised MEP cells in the bone marrow (Fig. 4b) and the prportion of double negative CD4−CD8− T cells in the spleen (Fig. 4c), which is consistent with a similar observation in the bone marrow of *Trichuris muris*-infected mice^49^. Intriguingly, other HSPC populations were positively correlated with adult worms and microfilariae (Fig. 3, Fig. 4, and Fig. 5). One possible explanation for the opposite direction of the correlations of parasite burdens with LSK, CLP, and MEP cells may thus be that HSPC cells typically respond to infection in a dose-dependent manner^50,51^, but that MEP cells specifically produce protective responses and/or that the parasites have evolved means to impede the differentiation of HSPC into MEP cells.

### Ageing predominantly affected adaptive responses to helminths

**Figure 6:**
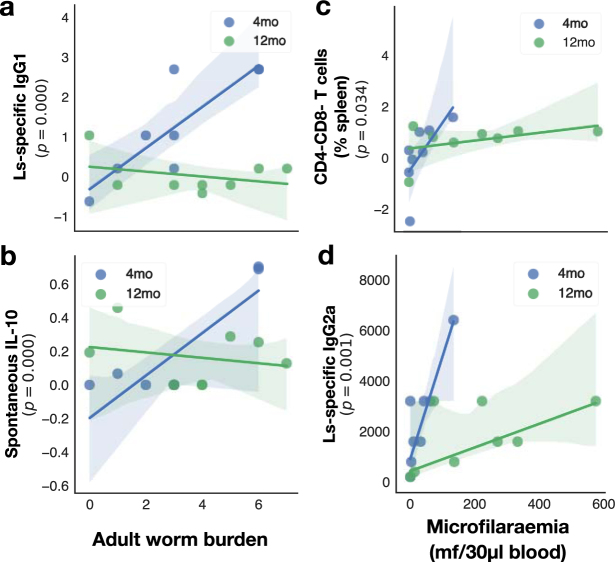
Age-dependent responses to adult worms and microfilariae. In 12mo mice, responses to increasing parasite burdens were weaker than in 4mo mice. Among the top features predicting adult worm burdens (see Fig. 3), (**a**) the concentration of parasite-specific IgG1 (P_AGExNW_ < 0.0001, GLM, n = 8 per group) and (**b**) the spontaneous production of IL-10 (P_AGExNW_ ≤ 0.0001, GLM, n = 8 per group) were the only features significantly affected by host age. Of the top features predicting circulating microfilariae densities (see Fig. 4), only (**c**) CD4-CD8- double negative T cells (P_AGE_ = 0.034, GLM, n = 8 per group) and (**d**) parasite-specific IgG2a (P_AGExMF_ = 0.001, GLM, n = 8 per group) were affected by host age. Each point represents a mouse, the solid line is the robust regression and the shaded areas represent the corresponding 95% bootstrapped confidence intervals.

We then sought to identify which immune factors among the best predictors of adult worm and microfilariae burdens, identified above, were significantly affected by mouse age. Thus, restricting our analysis to the top predictors of adult worm burden (29 features, Fig. 3a) and microfilarial density (26 features, Fig. 4a), we used each in turn as the response variable in a generalised linear model with age and the corresponding worm or microfilariae counts as explanatory variables, and selected those for which the interaction was significant. First, when examining predictors of adult burden, the concentration of parasite-specific IgG1 (Fig. 6a) and the spontaneous production of IL-10 (Fig. 6b) were the only features significantly affected by host age, with 4mo mice responding more strongly to increasing parasite doses than 12mo mice did. Similarly, of the top features predicting microfilariae densities (see Fig. 4), only CD4-CD8- double negative T splenocytes (Fig. 6c) and parasite-specific IgG2a (Fig. 6d) were reduced in 12mo mice compared to 4mo mice. The age-associated breakdown of parasite-specific IgG1 is of interest as this antibody, and the B cells that produce it, have been shown to be necessary for protective immunity against *L*. *sigmodontis*^21,24,46^. IL-10 has been linked to the suppression of immunity to filarial infections in this model^52,53^, suggesting 4mo mice may be better able to adjust their regulatory responses to parasite burdens than 12mo mice. This is consistent with our observation that 12mo were more susceptible than 4mo mice, and suggests that under chronic helminth infection, ageing attenuates the ability of the immune system to effectively respond to parasites dose^54^. Whether dampened adaptive immune responses are caused by a breakdown of the adaptive immune system or by a more efficient regulation of antihelminth immunity would require assessing their respective impacts on host fitness. However, our results are in line with previous studies showing that during primary infection, while adaptive immune responses do not affect the establishment and early survival of filarial parasites, it is essential for regulating their production of microfilariae^25,55–57^.

In conclusion, although we had predicted immunosenescence of the thymic and hematopoietic cell compartments to cause a collapse in the response to infection, our machine learning approach demonstrated that only a subset of each were reliably affected by age. The main correlates of protection against adult parasites, MEP cells in particular, were unaffected by age as were the other HSPC populations, suggesting both a role for MEP cells and their lineage in the regulation of filarial infections, and additional targets for prophylactic or therapeutic intervention against filariasis. The role of MEP cells in immunity to helminths remains largely unknown, and whether they are indeed protective and/or targets of parasite-driven immune suppression warrants further investigation. However, key adaptive immune effectors involved in the protective response against *L*. *sigmodontis*, IgG1 and IgG2a antibody, and naive T cells, were significantly reduced in the older hosts, and most likely explain the increased susceptibility to infection observed in that age class. Furthermore, these protective factors did not increase proportionally with parasite load in 12mo mice as they did in the younger animals, implying a loss of immune responsiveness or a stronger immune regulation with age. Together, these results underscore the power of machine learning to help disentangle complex biological processes and identify robust associations among the myriad of interactions taking place during infection. Our approach has allowed us to highlight the specific effects helminth infection imparts across the full maturation cycle of immune cells from HSPC within the bone marrow to effectors at the focal site of infection, within the same individual. As high throughput longitudinal datasets accumulate in the future, such approaches hold great promise for the development of individual-based predictive medicine and potential new interventions to help maintain quality of life across the life-course.

## Methods

### Ethics statement

All procedures on animals were approved by the University of Glasgow ethics committee and the UK Home Office (PPL60/4572), and immunity, ageing, and chronic helminth infection protocols conducted in accordance with the Animals (Scientific Procedures) Act 1986.

### Mice and infections

Thirty-two female BALB/c mice (Harlan UK) were allocated to a cross-factorial study design comparing age (2 levels) and helminth infection (2 levels) as follows. Mice were maintained by Harlan UK until they reached 2- and 10 months old. At 2 months, female BALB/c mice are immunologically and physiologically immature, while at 10 months they show advanced signs of thymic involution, i.e. at full immunological maturation, are skeletally fully adult and are at approximately half their typical median life-span^58,59^. After arrival in our animal unit, half the mice from each age group were infected subcutaneously with a single dose of 40 *L*. *sigmodontis* larvae in 0.2 ml RPMI1640 media, while controls received only control media. Mouse treatments were allocated randomly across individually-ventilated cages to minimise cage effects. The infection was left to progress naturally for 2 months, during which time larvae migrate to the pleural cavity, moult to the fourth larval stage one week post-inoculation (p.i.), moult once more one month p.i. to reach the adult stage, and then reproduce sexually and release microfilariae into the peripheral blood around 55 days p.i.^19^. The microfilariae remain in an arrested developmental state until they are taken up by a suitable arthropod vector in a subsequent blood meal; their detection and quantification is a standard diagnostic test for filarial infections and is indicative of the ability to transmit the disease. Mice were culled in two balanced experimental sessions three days apart by exsanguination under terminal anaesthesia followed by exposure to CO_2_-enriched atmosphere. All sampling was thus performed on 4 or 12-month-old mice, referred to hereafter as 4mo and 12mo mice, respectively.

### Parasite and tissue extraction and identification

Adult *L*. *sigmodontis* were flushed from the pleural cavity using 10 ml of cold PBS, and fixed in ~50 °C 70% ethanol for subsequent counting under a dissection microscope. Pleural lavage and pleural exudate cells were separated from the worms by allowing the worms to sediment naturally for 2 minutes, taking the cell-containing supernatant, and rinsing with fresh media. Cells were then prepared for cytokine assays and flow cytometry.

### *In vitro* stimulation of thoracic lymph node cells

Thoracic lymph nodes were harvested, and crushed in complete RPMI medium (10% FBS, 1% L-glutamine, 100 U/ml Penicillin-Streptomycin). For the antigen recall assay, 10^5^ lymph node cells were incubated with either 20 µg/ml of *L*. *sigmodontis* soluble antigen, µg/ml of anti CD3 antibody to force T cell proliferation (17A2/Rat IgG2b), or complete RPMI as a medium control, in triplicate. The supernatants were subsequently pooled together for cytokine quantification by ELISA.

### Antibody and cytokines quantification by ELISA

Concentrations of IL-4, IL-5, IFN-γ and IL-10 in pleural lavage fluid and lymph node culture assay supernatant were measured by sandwich ELISA. Plates were coated with either 0.06 µg IL-4 (clone: 11B11/Rat IgG1), 0.08 µg of IL-5 (TRFK5/Rat IgG1), 0.075 µg of IFN-γ (R4–6A2/Rat IgG1) or 0.2 µg of IL-10 (JES5–16E3/Rat IgG2b) capture antibody for 2 h at room temperature; plates were washed and 50 µl of the fluids were added for overnight incubation at 4 °C; plates were subsequently washed and biotinylated detection antibodies were added at either 0.013 µg for IL-4 (BVD6–24G2/Rat IgG1), 0.34 µg of IL-5 (TRFK4/Rat IgG2a), 0.05 µg of IFN-γ (XMG1.2/Rat IgG1) or 0.013 µg of IL-10 (JES5–2A5/IgG1). Plates were revealed with TMB-H2O2, and stopped using 1 M H2SO4 (1 M), and immediately read in a spectrophotometer at 450 nm. Specific anti *L*. *sigmodontis* IgG1 and IgG2a responses were measured in blood serum by indirect ELISA against whole soluble *L*. *sigmodontis* antigen extract coated overnight with 5 µg/ml of the antigen. Plates were washed and the serum added in duplicate doubling serial dilutions, and left overnight. Host IgG1 and IgG2a were detected with horseradish peroxidase (HRP)-conjugated antibodies. The reactions were revealed with TMB-H2O2 and stopped with 1 M H2SO4 and read at 450 nm. Titres were determined as the highest dilution factor for which O.D. values exceeded 3 standard deviations above control wells from the same plate.

### Pleural leukocyte identification and quantification by flow cytometry

Flow cytometry was performed using the FACS Canto flow cytometer (Becton Dickinson, Oxford, UK) and analysed using FlowJo software (Tree Star Incorporation, Oregon, USA). Pleural exudate cells were recovered from the pleural cavity using 10 ml lavage with cell culture medium, and plated out at 106 cells per staining combination. The cells were stained for macrophages using monoclonal antibodies specific for CD11b (clone: M1/70 / isotype: Rat IgG2b) and F4/80 (BM8/Rat IgG2a); eosinophils, for Siglec-F (E50–2440/Rat IgG2a); dendritic cells, for CD11c (HL3/Armenian Hamster IgG1) and MHCII (M5/114.15.2/Rat IgG2b); and T and B cells, for CD3 (17A2/Rat IgG2b), CD4 (GK1.5/Rat IgG2b) and CD19 (1D3/Rat IgG2a).

### Hematopoietic stem and progenitor cell identification and quantification by flow cytometry

Mouse bones (femur and tibia) were harvested, crushed in PBS + 2%FBS and filtered through a 0.7 µm mesh. Bone marrow (BM) cells ~10^6^ cells were stained with a lineage cocktail against biotinylated CD3, CD4, CD8a, Gr-1, B220, Ter-119, and CD11b and identified using streptavidin Pacific Blue™. Within the lineage negative fraction, stem/progenitor populations were assessed using antibodies against c-Kit and Sca-1 (Lin^−^c-Kit^+^Sca-1^+^ (LSK) and Lin^−^c-Kit^+^Sca-1^−^ (LK) cells). To assess the frequency of progenitor cells within the lineage negative fraction, c-Kit and Sca-1 staining, in addition to CD127, CD34 and CD16/32 was used to identify CMP (CD34^+^ CD16/32^−^), MEP (CD34^−^ CD16/32^−^), and GMP (CD34^+^ CD16/32^+^) cells^60^. All antibodies were purchased from BD Biosciences, Biolegend, eBioscience or Life Technologies. The percentage of various cell populations within the bone marrow (BM) was examined and the percentage of the parent gate and percentage of the total BM was reported (Supplementary Fig. S5). Cellularity was assessed (Hemovet®) and white blood cell count was multiplied by the white percentage positive cells within total BM to give absolute numbers per harvest. Flow cytometry analysis of myeloid and lymphoid mature cells was performed on BM, spleen, peripheral blood (PB) and thymi, which were prepared in PBS + 2%FBS and ~10^5^ cells were stained against markers of myeloid (Gr1, CD11b), lymphoid (CD19), erythroid (Ter119) or T cells (CD4 and CD8a). T cells were identified as naive or memory based on the cell surface staining of CD44 and CD45RB. PB was collected into EDTA-coated blood tubes (Sarstedt AG & Co) and stained with the appropriate antibody cocktail. After incubation, blood was lysed using EasyLyse (Dako UK Ltd) and subsequently analysed.

### Data Analysis

Our aim was to identify within the full maturation spectrum of immune cells the immune factors that best predicted (i) the age class (binary) of both infected and uninfected animals, (ii) the presence of infection by *L*. *sigmodontis* (binary), and (iii) worm and microfilariae burdens (continuous). Among the latter, we are also interested in those that were most affected by the ageing process (significance of the interaction term). Therefore, after using a principal component analysis to ascertain the presence of major age- and infection- associated variation among the immune variables we measured, we used a supervised learning approach to disentangle which variables were associated with age, infection presence, and infection burdens, and how they interacted. Because of the number of variables involved, some of which are likely to be highly correlated, and the limited number of samples available, we built models using machine learning algorithms that allow the identification and ranking of each immune variable in predicting the biological outcome of interest under those constraints. To ensure those models would generalise well, we repeatedly subsetted our data at random (10 rounds) applying a nested cross-validation scheme in which models were trained on 75% of the dataset using 5-fold stratified cross-validation, and the resulting optimised model predictions were tested against the ground-truth of the remaining 25% which the model had never seen before. Randomising which samples were assigned to each subset allowed us to test the influence of the allocations to each subset and to maximise the generalisability of our models while providing more robust inference of the immune mechanisms underlying responses to *L*. *sigmodontis* in 4mo and 12mo mice.

To identify the immune variables that best predicted age, infection, and parasite loads we compared the performance of common algorithms using their baseline (unoptimised) parameters (Supplementary Fig. S2). On our dataset, gradient boosting consistently performed as well as or better than the other approaches for the binary classification tasks of predicting age and host infection status (Supplementary Fig. S2a,b). Gradient boosting^33^, in its XGBoost (XGB) implementation^34^, is a sparsity-aware ensemble algorithm for supervised statistical learning that is trained in an additive manner, whereby shallow decision trees are built sequentially with each iteration minimising the error of the previous. Further, gradient boosting allows feature ranking to assess the relative contribution of each feature to the final trained model. Because XGB applies a non-parametric approach, we did not apply further parametric models to its model outputs. For regression tasks, Elastic Nets performed best in predicting adult worm and microfilariae numbers (Supplementary Fig. S2c,d). Elastic Nets perform particularly well for tasks where the number of variables exceeds the number of replicates, and tend to group together the variables that are highly correlated rather than selecting one over the others^36^. Correlation tests were then applied to the features that Elastic Net models weighted most to further assess their relationship with adult worm or microfilariae numbers.

The features we included in the models comprised 79 immune variables spanning the full differentiation cycle of immune cells: BM stem cells, thymic lymphocytes, PB leukocytes and antibodies, and cytokine concentrations at the site of infection and from *in vitro* restimulation of lymph node cells draining the site of infection (see full list in Supplementary methods). Random splits of this full dataset into test and training set were repeated 10 times, and within the training set, parameter optimisation was performed through exhaustive grid search and 10-fold cross-validation for each of the 10 repeated random splits. Feature importances, mean square errors (for regression models), and global and per-class accuracy metrics (for classification models) were recorded at each iteration and used to generate mean “bootstrapped” estimates ± standard errors of the mean (Fig. 2a, Fig. 3a, Fig. 4a, and Supplementary Fig. S4a). Only features exceeding 3 standard errors of the mean of all feature importances from the corresponding model were considered sufficiently robust and retained for further inference. Although XGBoost is not limited to linear relationships, to aid interpretation we plotted pairwise relationships between important immune features and continuous parasitological readouts as linear regressions (Fig. 3b–e and Fig. 4b,c).

Generalised linear models were then used to assess the effects of age and individual immune parameters on infection, and of age and infection on the immune features identified by XGBoost. Because mice were dissected on two consecutive days, experiment session was included as a fixed effect to account for uncontrolled variation in sampling dates, and normality of the models’ residuals ensured by transforming variables when necessary, and verified with the Shapiro-Wilk test for normality. To identify the immune features that both correlated with parasite counts and that were significantly affected by host age, we ranked the immune response variables using the p values of the linear interaction between age and parasite burden using a negative binomial Generalised Linear Model. Briefly, our algorithm iterated through each immune feature to calculate its p value with either microfilariae or adult worm counts as the response variable, and retained all features for which the p values of the interactive term were below 0.01. The Mann-Whitney U test was used for comparing parasite burdens between age classes. All statistical learning, linear modelling and corresponding figures were performed in Python packages scikit-learn, xgboost, statsmodels, matplotlib, seaborn, and pandas.

### Data availability

Data that support the findings of this study are available from the corresponding author upon request.

## Electronic supplementary material

Supplementary Information
