# Supplementary material for: Chronic helminth infection burden differentially affects haematopoietic cell development while ageing selectively impairs adaptive responses to infection
Source: Sci Rep. 2018 Feb 28;8:3802. doi: 10.1038/s41598-018-22083-5 (PMC5830876; doi:10.1038/s41598-018-22083-5)
Supplement: Supplementary file 1 — Supplementary Information [file 41598_2018_22083_MOESM1_ESM.pdf]

Chronic helminth infection burden differentially affects haematopoietic cell development while ageing selectively impairs adaptive responses to infection

Simon A. Babayan[a,*], Amy Sinclair[b], Jessica S. Duprez[a,c], Colin Selman[b,*]

[a]Institute of Biodiversity, Animal Health & Comparative Medicine, University of Glasgow, Glasgow, UK, and Moredun Research Institute, Pentlands Science Park, UK.

[b]Glasgow Ageing Research Network (GARNER), Institute of Biodiversity, Animal Health & Comparative Medicine, University of Glasgow, Glasgow, UK.

[c]School of Biomedical Sciences, University of Edinburgh, Edinburgh, UK.

*Corresponding authors

E-mail: simon.babayan@glasgow.ac.uk (SAB), colin.selman@glasgow.ac.uk (CS)

Supplementary information

Supplementary methods

Seventy-nine immune variables were included in the analysis:

Bone marrow: number of viable cells; Lineage negative (%); Lineage negative; LK progenitor (%); LK progenitor; LSK stem progenitor (%); LSK stem progenitor; 150+48- LT HSC (%); 150+48- LT HSC (%); 150-48- MPP (%); 150-48- MPP; GMP (%); GMP; CMP (%); CMP; MEP (%); MEP; CLP (%); CLP; Myeloid Gr1+CD11b+ (%); Myeloid Gr1+CD11b+; Erythroid Ter119+ (%); Erythroid Ter119+; B CD19+ (%); B CD19+; thymus: Viable; CD8+ (%); CD4+CD8+ (%); CD4+ (%); CD4-CD8- (%); CD4+ Memory (%); CD4+ Naive (%); (%) Memory; (%) Naive.

Spleen: number of viable cells; Myeloid Gr1+CD11b+ (%); Erythroid Ter119+ (%); B CD19+ (%); CD4-CD8+ (%); CD4+CD8+ (%); CD4+CD8- (%); CD4-CD8- (%); CD4 Memory (%); CD4 Naive (%); (%) Memory; (%) Naive.

PBMC: number of viable cells; Myeloid Gr1+CD11b+ (%); Erythroid Ter119+ (%); B CD19+ (%); CD4-CD8+ (%); CD4+ CD8+ (%); CD4+ CD8- (%); CD4- CD8- (%); CD4 Memory (%); CD4 Naive (%); (%) Memory; (%) Naive.

Pleural exudate: CD19+ cells; eosinophils; CD11c+ cells; MHCII+CD11c+ cells; macrophages; IL-5 concentration; IL-10 concentration; IgG1 titres; IgG2a titres; *in vitro* IL-4 media only; *in vitro* IL-4 α-CD3 stimulation; *in vitro* IL-4 Ls antigen stimulation; *in vitro* IL-10 media only; *in vitro* IL-10 α-CD3 stimulation; *in vitro* IL-10 Ls antigen stimulation; *in vitro* IFN-γ media only; *in vitro* IFN-γ α-CD3 stimulation; *in vitro* IFN-γ Ls antigen stimulation.

Supplementary figures

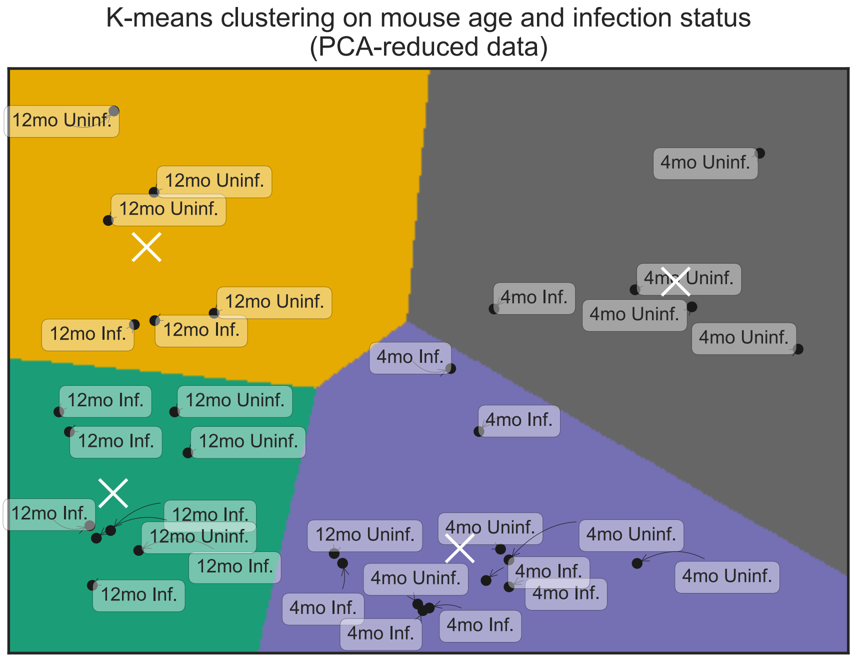

Figure S1: K-means clustering of immune features on mouse age and infection status. To visualise whether the immune systems of the mice differed between age groups and as a result of infection, we reduced the full set of 79 immune features to 2 dimensions using an unsupervised principal component analysis, and applied k-means clustering to identify clusters emerging from the resulting components (n = 32). While age classes separated well, the PCA was less able to distinguish infection status. 4mo: 4 month old mice; 12mo: 12 month-old mice; Inf: infected; Uninf: uninfected. Cluster centroids are marked with white cross and individual mice are marked with the black dot.

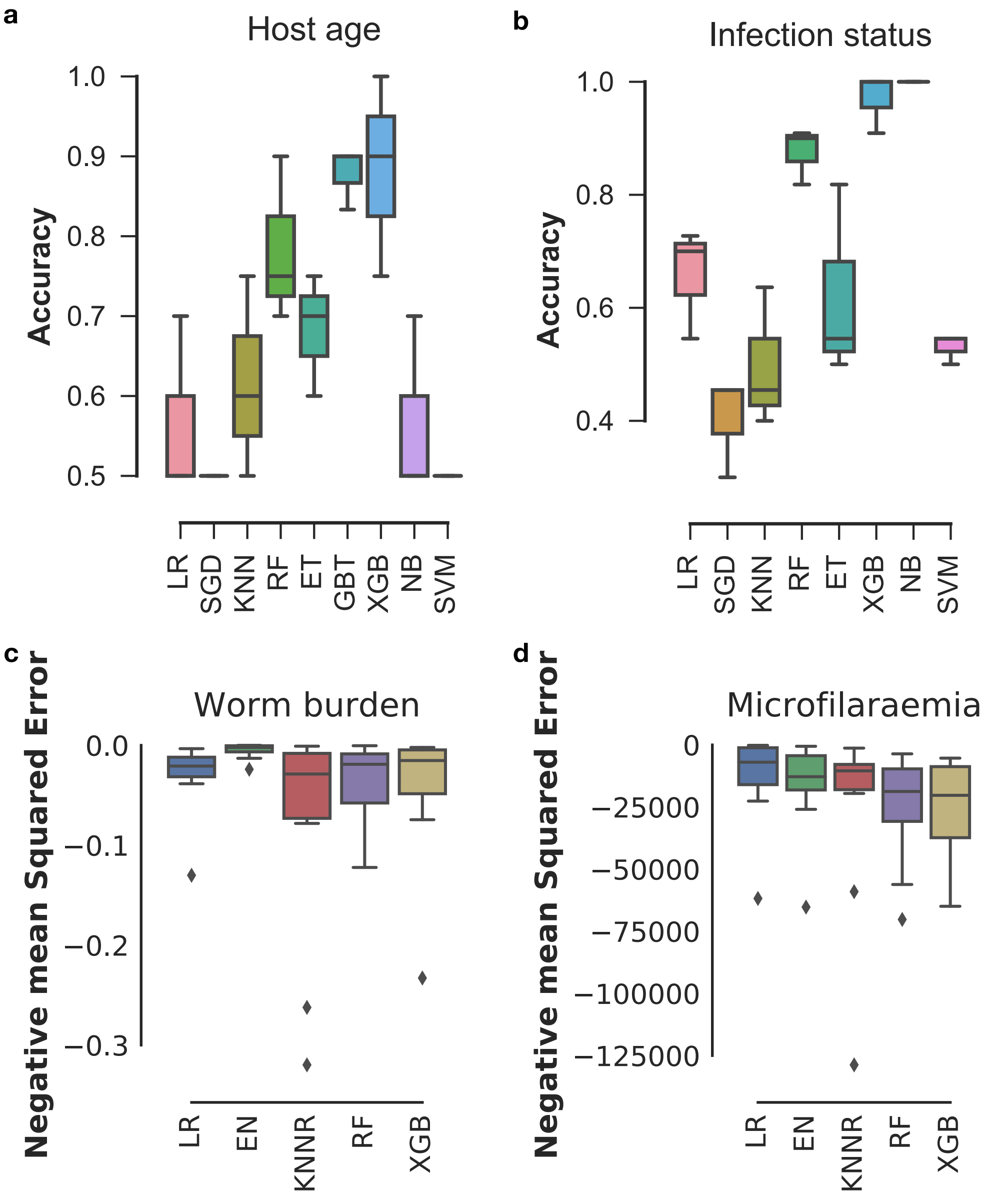

Figure S2: Baseline performance comparison of common machine learning algorithms. The accuracy of the most common machine learning algorithms was evaluated for the prediction of (a) host age, (b) infection status, (c) adult worm burden, and (d) microfilarial density in peripheral blood (n = 32 mice). For classification tasks ((a) and (b)), this included logistic regression (LR), stochastic gradient descent classifier (SGD), k nearest neighbours (KNN), random forests (RF), extra trees (ET), gradient boosting (XGB), naive Bayes (NB), and support vector machines (SVM). For regression tasks ((c) and (d)) this included multivariate linear regression (LR), Elastic Nets (EN), k nearest neighbours fr regression (KNNR), random forests (RF), and gradient boosting (XGB).

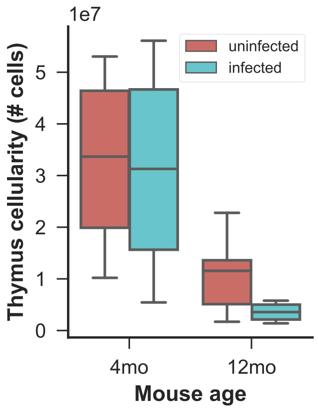

Figure S3: Thymic involution. The total number of cells in the thymus was lower in the 12mo mice than in the 4mo mice in both infected and uninfected mice ($P_{AGE} \leq 0.0001$, GLM, n = 8 per group).

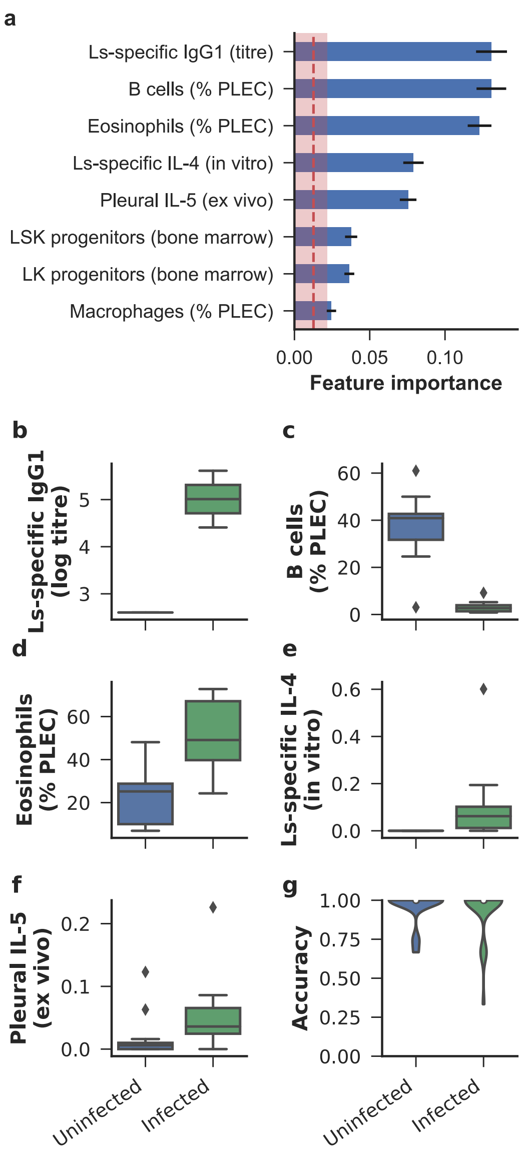

Figure S4: Immune variables predicting active infection. The importance of each immune factor in predicting the presence of an infection irrespective of age (n = 16 in each group) was estimated by measuring the average gain in the purity of daughter nodes when decision tree splits that used that feature for classifying mice as infected or uninfected. We used a cut-off of 3 standard errors (red-shaded zone) above the mean (red dotted line) of all importances below which features were not considered robust enough for further inference. (a) Top predictors of infection status included parasite-specific responses from the adaptive immune system and the cellular composition at the site of infection, allowing the model to achieve 100% prediction accuracy. Horizontal bars represent means and associated errors of the feature importances generated from 10 models trained on different randomised 75%/25% train/split repeats. Top predictors of infection status included the serum concentrations of *L. sigmodontis*-specific (b) IgG1, (c) the proportion of B cells and (d) of eosinophils within PLEC, (e) the production if IL-4 by T cells stimulated with *L. sigmodontis* antigen *in vitro* and (f) the concentration of IL-5 in the pleural cavity. (g) shows a violinplot of the distribution of per-class accuracies of the 10 XGB models. In boxplots, horizontal lines represent the group median, boxes the interquartile range, whiskers the overall range, and points represent outliers (< ±1.5 x the interquartile range).

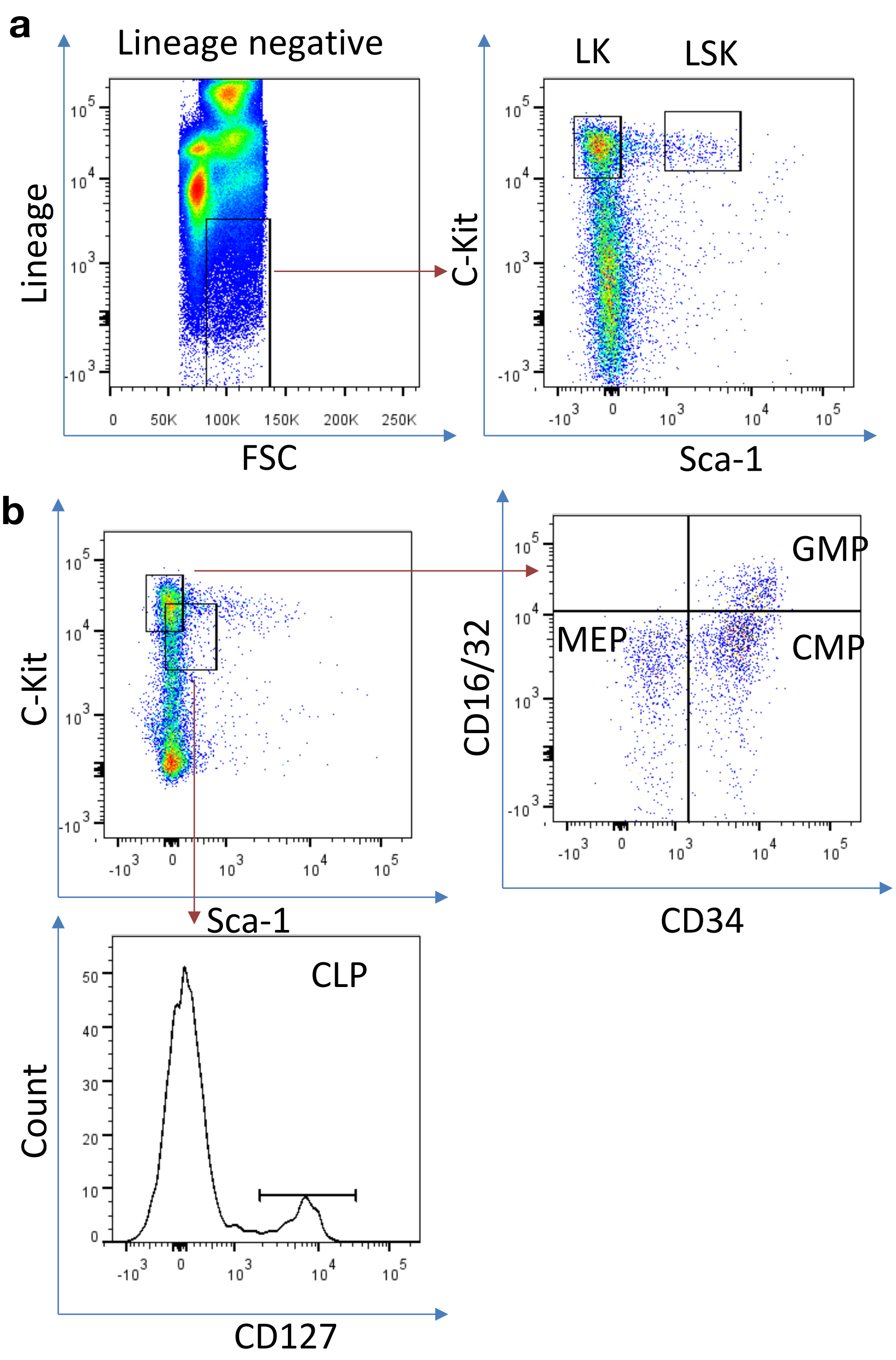

Figure S5: Flow cytometry gating strategy. Viable BM cells were identified using forward and side scatter outputs of a flow cytometry analysis. Doublets were excluded using forward scatter area versus forward scatter width. (a) Cells negative for antibodies in the lineage cocktail were identified and within this population cells were identified as stem (LSK) or progenitor (LK) based on their expression of Sca-1 and c-Kit. (b) Within the LK gate, MEP, CMP, and GMP cells were identified using CD16/32 and CD34. A c-Kit^{med} and Sca-1^{med} gate was selected to identify the CLP population using CD127.
